# Learners’ perspectives on training for HIV management in sub-Saharan Africa: Insights from the AFREhealth HIV project

**DOI:** 10.4102/phcfm.v17i1.4789

**Published:** 2025-10-24

**Authors:** Manoko Lediga, Ian Couper, Shayanne Martin, Michael Reid, Edward Dassah, Miliard Derbew, Marietjie de Villiers, Maeve Forster, Onesmus Gachuno, Clara Haruzivishe, Abigail Kazembe, Keneilwe Motlhatlhedi, Nisha Nadesan-Reddy, Catherine Ngoma, Georgina Odaibo, Fatima Suleman, Deborah von Zinkernagel, David Sears

**Affiliations:** 1 Division of Rural Health (Ukwanda), Faculty of Medicine and Health Sciences, Stellenbosch University, Cape Town, South Africa; 2 Institute for Global Health Sciences, University of California, San Francisco, United States of America; 3 Department of Medicine, Division of Infectious Diseases, University of California, San Francisco, United States of America; 4 School of Public Health, Kwame Nkrumah University of Science and Technology, Kamusi, Ghana; 5 School of Medicine, Addis Ababa University, Addis Ababa, Ethiopia; 6 Division of Family Medicine and Primary Care, Faculty of Medicine and Health Sciences, Stellenbosch University, Cape Town, South Africa; 7 Department of Obstetrics and Gynaecology, University of Nairobi, Nairobi, Kenya; 8 Faculty of Medicine and Health Sciences, University of Zimbabwe, Harare, Zimbabwe; 9 Faculty of Health Sciences, Kamuzu University of Health Sciences, Lilongwe, Malawi; 10 Faculty of Medicine, University of Botswana, Gaborone, Botswana; 11 School of Nursing and Public Health, University of KwaZulu-Natal, Durban, South Africa; 12 School of Nursing Sciences, University of Zambia, Lusaka, Zambia; 13 Department of Virology, College of Medicine, University of Ibadan, Ibadan, Nigeria; 14 School of Health Sciences, University of KwaZulu-Natal, Durban, South Africa; 15 Division of Infectious Diseases, University of California, San Francisco, United States of America

**Keywords:** HIV management, interprofessional collaboration, AFREhealth HIV, healthcare professionals, sub-Saharan Africa

## Abstract

**Background:**

The African Forum for Health Education and Research human immunodeficiency virus management training (AFREhealth HIV) project was launched in 2019. The project offers a reimagined model for interprofessional training and mentorship to improve clinical care and equip healthcare workers with the technical knowledge and clinical tools to respond to HIV and other health issues.

**Aim:**

The study aims to evaluate learners’ experiences of interprofessional health workforce capacity building across sub-Saharan Africa (SSA) to enhance HIV management.

**Setting:**

Participants included pre-service medical and nursing students and early career professionals (learners). Learners were associated with 14 AFREhealth partners in 11 SSA countries.

**Methods:**

Learners attending AFREhealth HIV training workshops were invited to provide feedback using a standardised online form, which included 28 Likert-type questions and 3 open-ended questions. Analysis of the 3 open-ended questions was done by coding responses into a set of common themes and sub-themes.

**Results:**

Findings showed that of the 3711 learners who participated, only 2570 completed the post-training evaluation. Findings also showed that the learners appreciated the approach adopted in the workshops and believed they gained significant knowledge and skills for themselves. The importance of collaborative, team-based and interprofessional approaches throughout the training was highlighted.

**Conclusion:**

The training approach adopted by the AFREhealth HIV project has proven to be highly effective. The project has thus continued to target final-year health professional students and working health professionals at affiliated training sites, with module workshops being offered both online and onsite.

**Contribution:**

Collaborative and interprofessional approaches to training health professionals for HIV management can improve knowledge, skills and, very importantly, attitudes, with the potential thus to improve the quality of team-based care provided especially in low-resource settings.

## Introduction

Human immunodeficiency virus and acquired immunodeficiency syndrome (HIV and AIDS) continues to be one of the leading causes of the global burden of disease.^1^ Although the impact of the epidemic continues to vary considerably among countries and regions, sub-Saharan Africa (SSA) carries a disproportionate load of the disease.^2^ The World Health Organization (WHO) data show that 25.6 million or 70% of all people living with HIV live in the region.^3^ Despite significant advances in managing HIV over the years, there are still inconsistencies in the engagement of healthcare workers with HIV care resulting in unmet needs in HIV management.^4^ Managing HIV presents unique challenges that require specialised knowledge and approaches. Most research on HIV management has focussed on clinical data, leaving much to be learnt about non-clinical correlates.^4^ In the face of these needs the African Forum for Health Education and Research human immunodeficiency virus management training (AFREhealth HIV) project was launched in 2019 as a collaboration between the AFREhealth and fourteen training institutions across Africa. The AFREhealth HIV management training project offers a reimagined model for interprofessional training and mentorship to improve clinical care and equip clinicians with the technical knowledge and clinical tools to respond to HIV, coronavirus disease 2019 (COVID-19) and other health issues.^5^

The AFREhealth HIV project identified healthcare professionals (HCPs) as key players in the efforts towards managing HIV as a global health problem.^6^ The management of patients living with HIV and AIDS requires HCPs to practice evidence-based medicine. Adequate training of pre-service HCPs in the care of patients with HIV and AIDS and a well-developed system of ongoing professional development are thus needed to ensure that HCPs remain up to date as the understanding of HIV and its management constantly evolves.^5^

As a result, 17 training modules were developed to focus on HIV management, quality improvement and collaborative practice (see Table 1). All modules were developed by AFREhealth and a team of expert advisors, including local and international HIV and education experts. The AFREhealth HIV project partnered with local health professional training institutions to conduct training on HIV management. Using a subset of the developed modules, these local AFREhealth HIV partners facilitated interprofessional, small group, case-based learning, which targeted pre-service and early career professionals across SSA during 2-day interactive workshops. The frequency of training offered, ratio of learners to facilitators, a mix of cadres and course timing were all determined by AFREhealth partners.

**TABLE 1:** Topics Covered in the Course.

Topics	Examples
Managing common co infections	Infections such as TB, Pneumocystis Pneumonia, Cryptococcal meningitis, sepsis.
Diagnosis of HIV	Diagnosing HIV in different risk groups, genders and ages.
Management of people living with HIV	Initiating antiretroviral therapy (ART); adherence; supporting pregnant women.
Identifying and managing treatment failure	Virologic failure; traditional and complementary medicine
Preventing acquisition of HIV infection	Pre-exposure and post-exposure prophylaxis (PrEP & PEP); patient education.
Preventing and managing HIV infections in children and adolescents	Prevention of mother-to-child transmission (PMTCT); paediatric HIV infection; care for patients with Perinatally-Acquired HIV.
Other common conditions associated with HIV infection	Hypertension; diabetes; non-communicable diseases (NCDs).
Care for populations at high risk for HIV infection	Men who have sex with men; women of childbearing age.
Improving quality of care	Quality improvement; teamwork.
Supporting health systems needs for HIV service delivery	Addressing broader health systems requirements; health workforce challenges; community-based care delivery.

HIV, human immunodeficiency virus; TB, tuberculosis.

Since 2010, there have been several calls for changes in the way HCPs are trained.^7,8^ In response, there have been a number of ongoing developments in Health Professions Education, including competency-based education, interprofessional education and the large-scale application of information technology to education.^9^ Recent research highlights how optimised team-based approaches to healthcare training can improve the quality of care provided.^10,11^ These all influenced the content and structure of the AFREhealth HIV training modules and workshops, which incorporated topics that were appropriate for the context and the profession involved. These workshops included discussions on interprofessional teamwork and collaboration, based on patient case scenarios, addressing issues such as shortage of different cadres of health workers, resource constraints, hierarchy and team dynamics, across various modules.

An evaluation of the AFREhealth HIV training project was conducted to assess the impact of this interprofessional case-based training programme on HIV clinical knowledge and confidence among health professionals working in high HIV-burden settings in SSA. The quantitative component of this evaluation has been published elsewhere and showed that the training intervention was associated with significantly greater knowledge scores and confidence levels for all learners, regardless of health profession.^5^ This article focusses on the qualitative component of this evaluation and highlights participants’ experiences of small group interprofessional training for HIV management in Africa. Understanding these experiences offers insights into why learners’ knowledge and confidence improve, what components they found particularly valuable and how such a learning process can support and reinforce team-based HIV management to improve the care of people living with HIV in Africa.

## Research methods and design

### Study design

The study was part of a larger study that focussed on evaluating a HIV management training programme. This article reports on the qualitative findings. Training was conducted by 14 AFREhealth partners in 11 countries in SSA. The evaluation included pre- and post-tests, completed by learners before and after each module, measuring their knowledge and confidence for that module. The training evaluation also included a post-training assessment, completed by learners after they had completed all modules included in their training. Here we analyse the post-training evaluation responses of all learners who had completed both their module pre- and post-tests.

All learners who participated in the workshops were invited to complete the post-training evaluation by each AFREhealth collaborating institution that delivered the training.

### Data collection

Post-training evaluation for the trainings that were conducted between 01 October 2019 and 31 March 2020 was collected. The post-training evaluation included three open-ended questions regarding learners’ experiences of the training. The questions asked learners to reflect on their learning experiences during the workshops in relation to what they would do differently in terms of interprofessional collaboration, quality improvement and HIV and AIDS care in their workplaces. Only learners who completed the post-tests were included in this study because the evaluation questions were included as part of these post-tests.

### Analysis

Thematic content analysis was used to analyse the qualitative data. Responses across all workshops were organised into a set of common themes. Coding of the data was carried out inductively in MS Excel by two members of the team (M.L. and I.C.) using an iterative process of repeated reading of the responses and discussion, leading to a process of coding cascades up from codes through to categories, sub-themes and themes. Key themes were identified by continuously cross-reading transcripts, evaluating patterns and exploring relationships. The themes were then organised in an Excel spreadsheet, with data linked to the theme and categories and shared with the full research team, which reflected on the original data. Authors representing all the collaborating institutions, reviewed the results and confirmed that their learners’ views were accurately represented in the final themes.

### Ethical considerations

Ethical approval to conduct this study was obtained from the University of California, San Francisco’s Institutional Review Board (IRB) in San Francisco, California (No. 19–28 447). Verbal consent was obtained from participants at the time of the evaluation for use of anonymised data in subsequent research. Completion of the online post-tests, including the evaluation question, required participants to click an informed consent button to indicate agreement for use of the data in research.

## Results

### Demographics of learners

A total of 157 workshops were conducted and included 3711 learners. Learners included nurses and/or midwives medical doctors, pharmacists and laboratorians (in descending numerical order). Some learners indicated that they were from ‘other’ fields within the health sciences, but those fields were not specified (see Table 2).

**TABLE 2:** Learners’ demographics (*N* = 2570).

Demographics	Number of learners who completed reflections	%
**Gender identity**		
Male	1091	51
Female	1298	42
Unspecified	181	6
**Training level**		
Preservice student	1463	57
Postgraduate (new provider within 12 months of graduation)	559	22
Postgraduate (beyond 12 months after of graduation)	548	21
**Profession category**		
Nursing and/or midwifery	1084	42
Medical	725	28
Pharmacy	317	12
Laboratory	237	9
Unspecified	207	8

**FIGURE 1:**
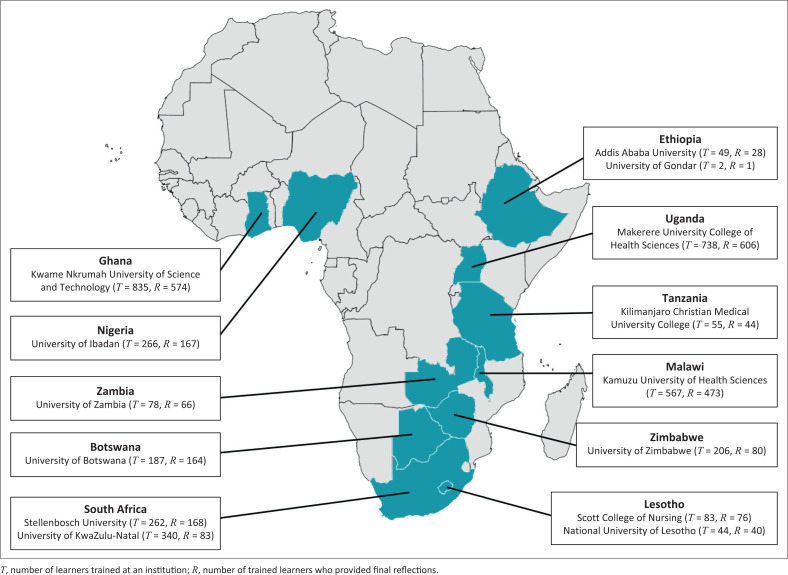
Universities and countries of origin of learners.

A majority of learners were from Kwame Nkrumah University of Science and Technology, Ghana and Makerere University College of Health Sciences, Uganda. Learners’ universities and countries are shown in Figure 1.^11^

### Identified themes

Three main themes were identified: value of training, communication and value of teamwork. In this section quotations are coded according to gender, career stage, that is, pre-service (student) or working professional (graduate) and profession.

#### Theme 1: Value of training

The first main theme identified was the value of the training, which included sub-themes about interprofessional collaboration, broadened HIV knowledge and building relationships. The modules were seen to offer great value for the learners and allowed them to see and think beyond their previous understanding.

Learners’ discussions highlighted three key sub-themes in relation to the value of training: interprofessional collaboration, broadened HIV knowledge and building relationships.

**Interprofessional collaboration:** Interprofessional collaboration was recognised as important by all those that were involved in the course. They highlighted how interprofessional learning broke down silos and opened a new door for engagement with HCPs from different backgrounds. They mentioned having learnt a lot from the curriculum and each other that helped them to appreciate professionals in other fields. Ultimately, learners were encouraged to make health systems improvements as some of the learners saw this kind of collaboration to be necessary as it taught them to put patients in the centre of everything:

‘Interprofessional collaboration is the core of effective healthcare system. Holistic approach to a patient … encourage collaboration. Having good interpersonal relation with co-workers promote … interprofessional collaboration too.’ (Male, Graduate, Medicine, 307)

‘I will collaborate with other health professionals who are my colleagues. If it’s in the best interest of our patient. I will respond their opinion & clinical Judgement, and I will tell them my conclusion respectively.’ (Male, Student, Pharmacy, 366)

‘It will be much easier for me to collaborate with other disciplines since we now more clear about their role.’ (Female, Student, Medicine)

‘I have understood better how to address so many issues that are associated with the management of PWH and this will improve how I care for my patients.’ (Female, Graduate, Medicine, 136)

‘By involving all professions in the health care system to make complex decisions for the betterment of the patient.’ (Male, Student, Pharmacy, 5)

‘By putting all that was taught into practice especially by ensuring interprofessional collaboration among health workers whilst putting the patient at the centre as the major stakeholders in the health sector.’ (Female, Student, Medicine, 136)

‘Try as much as possible to have different health professions when dealing with any problems mapping solutions together.’ (Female, Graduate, Pharmacy, 42)

‘I think the course was excellently structured, it forced the different sectors to work together to solve issues which also exposed various knowledge gaps within those sectors. It would now better assist workplace collaboration as we take back these gaps.’ (Male, Graduate, Medicine, 18)

‘The training … improved my understanding of the several ways HIV patients can be managed in a team-based approach.’ (Male, Student, Laboratory Science, 143)

**Broadened human immunodeficiency virus knowledge:** Learners were asked what they thought about the course. They mostly mentioned how the training enhanced their knowledge of HIV care. The training programme was viewed as having a positive impact on their ability to deliver HIV care:

‘From the training I understood that they are more dynamics to HIV patients that are not covered in my profession, the training provided a platform that made me aware of knowledge that is very significant to know.’ (Female, Student, Unspecified profession, 65)

‘This training program has helped me consolidate my knowledge! It will be beneficial for my patients and me as I would be giving them care with my maximum capacity.’ (Female, Graduate, Medicine, 25)

‘It will help health professionals know how to properly handle HIV patients and reduce other infections.’ (Female, Student, Nursing and/or Midwifery, 14)

‘It will improve their health because I am going to use my knowledge I learned to their better health.’ (Female, Student, Medicine, 4)

‘I believe, I have gained due knowledge and essence of practice to manage patients better than earlier.’ (Male, Graduate, Medicine, 37)

‘It broadened my knowledge outside of the unit I work into a broader spectrum of care.’ (Female, Graduate, Nursing and/or Midwifery, 4)

**Building relationships:** Learners described acquiring a better approach to relationships with patients, individually and as a team. They also appreciated the training because it allowed them to improve their relationships with other health professionals:

‘Think more holistically and have better approach to doctor patient relationship and discussions.’ (Female, Student, Medicine, 2)

‘I would consider rendering help to patients together with lab technicians, clinicians, fellow nurses and other stakeholders at my sphere of work so that together we can help our clients in the hospital.’ (Female, Graduate, Nursing and/or Midwifery, 37)

‘Construct good relations with other health professionals.’ (Female, Student, Laboratory services, 3)

#### Theme 2: Communication

The second main theme was communication. The learners commended the training for opening new channels of communication between health workers from different professions, something that rarely happened in their experience as they had mostly worked independently. The following sub-themes were identified: effective communication around patient care, the importance of giving feedback and feeling empowered to pass on learning content.

**Effective communication around patient care:** The learners highlighted how the training would impact the way they communicate with and care for patients. They acknowledged the importance of communication with colleagues in order to improve patient care. They also mentioned how the training gave them the confidence to approach HIV-related issues. It was noted that there is still a lot of stigmas around HIV, even among healthcare workers. Many learners mentioned their struggles of talking about HIV with HIV patients but felt this training had improved that. Some learners mentioned how this training made them have empathy and compassion towards persons living with HIV. Through this communication, some mentioned becoming familiar with drugs their patients were receiving.

‘It is going to improve my care of patient in terms of communication skills and how to give counselling to promote adherence.’ (Male, Student, Nursing and/or Midwifery, 14)

‘More knowledge about the disease has been given and proper communication with other professionals would help.’ (Female, Student, Medicine, 803)

‘… [*I*]t gave me confidence on how to approach HIV related issues in children without any worries because honestly I have always been scared of working with children especially infants. so, after this session I’m confident I will try to overcome my fear.’ (Female, Student, Nursing and/or Midwifery, 58)

‘It will make me more comfortable and help me to deal very well with caring for people living with HIV and AIDS.’ (Female, Student, Medicine, 29)

‘It will help me to have empathy when I’m taking care of my patient as well as communicating with my patients in a non-judgemental manner.’ (Female, Student, Nursing and/or Midwifery, 4)

‘I came to realise that there is much more for me to do with regards to getting to know or be familiar with existing drugs that are prescribed to my patients as a counsellor. The only way to really get to know their effects and benefits is my interacting with patients.’ (Female, Student, Unspecified profession, 15)

**The importance of sharing information:** Learners valued the training and wanted to pass on the knowledge they had acquired by sharing information with their colleagues. Furthermore, sharing knowledge was seen to be important in order to provide the best patient care:

‘I will give feedback and to insist them [*colleagues*] to practise together more in order to reach 95/95/95 goals.’ (Unspecified gender, Student, Nursing and/or Midwifery, 206)

‘I will be going to conduct feedback to my fellow staff about [*AFREhealth*] HIV.’ (Unspecified gender, Graduate, Nursing and/or Midwifery, 24)

‘By sharing all the information with the colleagues as to provide the best quality care.’ (Female, Graduate, Nursing and/or Midwifery, 63)

#### Theme 3: Value of teamwork

The last theme was the value of teamwork that occurred during the workshops. The learners appreciated learning in teams during the course as it granted them an opportunity to work with colleagues they would not necessarily engage with on day-to-day basis.

Learners mentioned how working in teams improved the quality of their work and how they engaged with each other. The learners valued the course because it demonstrated the value of the team working together, each with their own role.

Some learners mentioned that because of AFREhealth HIV training, they want to start following WHO guidelines properly, which requires better teamwork:

‘Learn a lot, especially teamwork, improving quality in a team and general medical practice work I have acquired for patients benefit.’ (Male, Student, Pharmacy, 11)

‘… The teamwork and spirit established during the course of the training will be continued by us the privileged trainees outside the confines of the training.’ (Unspecified gender, Graduate, Medicine, 134)

‘They encourage teamwork and lots of participation. Also inform fellow health care professionals that no one is more important than the other. We are all important and play huge role in ensuring that the patient gets back to their normal life and feels better.’ (Unspecified gender, Student, Pharmacy, 234)

‘By handling a patient as a team of specialists hence all patient problems will be tackled and handled.’ (Female, Graduate, Unspecified Profession, 458)

‘By encouraging teamwork and following the WHO guidelines.’ (Male, Student, Medicine, 307)

‘After the workshop I am now aware that the entire health team is essential in treatment of HIV, and they all play vital roles therefore no profession is better than the other …’ (Female, Student, Nursing and/or Midwifery, 22)

## Discussion

This article presents how a large multi-country interprofessional training for HIV management broadened HIV knowledge and encouraged interprofessional collaboration, relationship building between cadres, effective communication around patient care and giving feedback. The training included learners from 14 health professions training institutions across SSA. While a majority of the learners were at pre-service level, a substantial number of post-service professionals were also part of the training.

Healthcare professionals remain essential for optimisation of the HIV response; however, managing the healthcare needs of HIV patients presents unique challenges that require specialised knowledge and approaches.^12^ Research shows that managing HIV is particularly challenging in low resources settings.^13^ The role of HCPs in managing HIV is critical, and exploring their experiences can help improve care and management. In addition, HCPs need continuous training, education, and professional development to remain abreast of the latest advancements in research, innovative technologies, treatment methods, guidelines and evidence-based practices.^14^ This includes adapting to regulatory alterations to improve clinical competency, support personal and professional advancement and provide patient-centred care.^15^

One of the challenges related to knowledge are widespread stigmatising beliefs and attitudes that are well documented among HCPs.^16^ These challenges interfere with preventative care and management, creating barriers to patients seeking information, testing and medical care.^17^ Most of the learners in this study mentioned having preconceived ideas about what HIV is and how that overshadowed their ability to care for HIV patients. These ideas were challenged in the training; learners mentioned how the training broadened their knowledge and understanding of HIV. Human immunodeficiency virus-related stigma continues to be a significant human rights issue, and tackling HIV-associated stigma and discrimination is essential to reach the objective of eradicating HIV and AIDS.^18^ Addressing the issue of stigma directly through open discussions in interprofessional training workshops with peers offers a way to assist in reducing stigma and to support HCPs to reflect on their own attitudes so that they can become role models in their communities.^19^

The importance of collaborative, team-based and interprofessional approaches throughout the training were highlighted by the learners. They mentioned how such interprofessional approaches contributed to improvements in knowledge and clinical confidence. Studies have shown that collaborative care results in improved patient identification, accessibility to available resources and referral,^20^ especially in the treatment of multimorbidity that requires the involvement of different professions.^21^ Collaboration among physicians, nurses and other relevant HCPs is particularly important in HIV management, so it is encouraging that these were specifically highlighted as important areas of development by learners. The results thus underscore the importance of interprofessional approaches to learning and how they support HIV training programmes.

The learners valued the communication that occurred during the training. Communication among HCPs has proven to impact care for patients. It was noted that there are still a lot of stigmas around HIV, even among healthcare workers. Collaboration is only as effective as the communication among team members. When HCPs are not communicating effectively, patient safety is at risk for several reasons,^22^ especially where HIV management is concerned, as it may lead to misinterpretation of information. The learners valued the chance to practice communicating with each other across professions as part of their training.

The training was also seen to be particularly significant in the context of the limited resources available to support practicing HCPs in SSA.^5^ Because of the nature of the training, the learners highlighted how they immersed themselves in their discussion groups and how much they learnt from HCPs from different fields than their own. As a result, they wanted to work more closely with HCPs from different fields, which they indicated is not common in their workspaces. They saw the value of working together to support each other in light of human resource constraints and other system limitations. This emphasis on effective teamwork is recognised as an essential tool for constructing a more effective healthcare delivery system.^23^

The learners thus valued the approaches adopted in the workshops and believed they had gained significant knowledge and skills for themselves, which they wanted to pass on to others. They expected that this would lead to an improvement in the quality of the patient care they deliver, which accords with evidence that respecting and meeting patients’ and caregivers’ needs are essential for good healthcare services.^24^ Overall, training was felt to positively impact the knowledge and confidence of learners. Moreover, the training programme successfully leveraged an extensive network of training institutions across SSA to deliver high quality, standardised training, while allowing for contextual adaptation and flexible approaches to the delivery of modules based on local situations. There is great potential for this approach beyond HIV in terms of team-based training to improve the quality of care more broadly. This model was subsequently implemented during the pandemic for online training of healthcare teams to respond to COVID-19, and team-based online HIV management training workshops using adaptations of the AFREhealth HIV modules were also conducted successfully.^25^ It is thus recommended that the use of a similar approach to assist the interprofessional health workforce in responding to other current and future health challenges should be explored.

### Strengths and limitations

The strengths and limitations of the study are as follows:

- This was a large cohort study. It included over 3000 learners from different countries in the SSA region.
- As there were not a wide range of health professionals in the workshops, the findings were weighted towards nurses/midwives and doctors. This might have influenced the responses we received.
- Because of limitations in Internet connectivity at some training sites, not all learners were able to complete the pre-assessments, thus inhibiting students from completing the post-assessments. The study size was therefore reduced from 3711 to 2570 learners.
- As data were collected in 2020, the analysis does not provide insights into whether the training led to improvements in clinical care.
- The researchers would like to acknowledge that the use of English meant there was a bias in favour of anglophone respondents, as opposed to francophone and lusophone representations.

## Conclusion

The training approach adopted by the AFREhealth HIV project has proven to be valuable not only because of the knowledge gained in relation to HIV care associated with it^5,25^ but also because of the broader learning that occurred with teamwork, communication, relationships and interprofessional collaboration, which have the potential to have an even greater impact on patient care and service delivery in general. The AFREhealth HIV management training project has continued to target final-year health professional students and working health professionals at affiliated training sites, with module workshops being offered both online and onsite.
